# Reactivation of Herpes Zoster Virus After COVID-19 Vaccination: Is There Any Association?

**DOI:** 10.7759/cureus.25195

**Published:** 2022-05-21

**Authors:** Surbhi Agrawal, Kapila Verma, Ishan Verma, Jagriti Gandhi

**Affiliations:** 1 Dermatology, LN Medical College and Research Center, Bhopal, IND; 2 Dermatology, LN Medical College and JK Hospital, Bhopal, IND; 3 Medicine, LN Medical College and Research Center, Bhopal, IND

**Keywords:** covidpandemic, herpes, covid19 vaccine side effects, covid-19 vaccination, herpes zoster reactivation

## Abstract

SARS-CoV-2 disease, COVID-19 infection, is a multi-system illness that has afflicted people all over the world. A number of vaccines have been produced to combat the current COVID-19 pandemic, and a variety of side effects have been recorded following the vaccination. However, there are limited data on the negative effects of immunological reactivation following vaccination. We report 10 incidences of herpes zoster reactivation within 7-21 days of getting the COVID-19 vaccination. Transient immunomodulation following vaccination, similar to that seen in COVID-19 illness, could be one explanation for this reactivation. These cases highlight the significance of continuing to examine vaccine safety during the COVID-19 pandemic's ongoing mass vaccination campaign. We also underline the importance of peripheral health professionals in the management and reporting of any vaccination-associated adverse event.

## Introduction

In view of the current COVID-19 pandemic, World Health Organisation (WHO) issued the first emergency use for the COVID-19 vaccine in December 2020 [1]. Various types of vaccines have been tried, including DNA- and RNA-based, non-replicating and inactivated with variable authorization all over the world. In India, two vaccines are now in use to combat the spread of the severe acute respiratory syndrome coronavirus 2 (SARS-CoV-2): ChAdOx1-nCOV-19 (Covishield™) and BBV-152 (Covaxin®) [2]. Vaccines are essential to protect those who are at high risk of complications and to create herd immunity, which could help limit disease outbreaks. Side effects of COVID-19 vaccines have been mostly mild to moderate, including fever, fatigue, body ache, headache, chills, muscle pain, diarrhea, and skin rashes [3]. However, the surveillance of long-term side effects and rare safety concerns associated with the COVID-19 vaccine is still ongoing. One such rare complication after the COVID-19 vaccination is herpes zoster reactivation. Certain proposed mechanisms are decreased counts of CD3+ CD8+ lymphocyte and CD4+ T cells functional impairment [4]. Further research to understand the underlying mechanisms for herpes zoster virus reactivation after COVID-19 vaccination needs to be carried out.

Varicella zoster virus (VZV) is a commonly encountered infectious disease in dermatology outpatient departments (OPDs) presenting as varicella/chickenpox or herpes zoster. VZV, a DNA virus of the Herpes family, is a neurotropic virus that causes chickenpox as a primary infection, which is a relatively mild, self-limiting childhood illness with a characteristic exanthem, following which VZV remains dormant in dorsal root ganglion for life. Upon reactivation, there is anterograde axonal migration of the virus to the periphery, causing a characteristic painful dermatomal rash with unilateral involvement, which is known as herpes zoster or shingles [5]. Reactivation is more common in the older population, most commonly in individuals aged 60 and above, as a result of diminishing cell-mediated immunity that occurs with age, as well as other factors that contribute to a compromised immune system [6]. Certain other triggering factors, such as vaccines, drugs, stress, aging, and the natural decline of immunity, are also seen to be responsible for the reactivation of VZV. Paraesthesia is the first symptom of shingles, followed by a rash or blisters that crust, scab, and get cured over the next 7-10 days. Approximately 20% of patients develop post-herpetic neuralgia, usually lasting for months to years [7].

We present a study of patients who developed reactivation of VZV after receiving the Covishield vaccine to fight against SARS-CoV-2. Few cases in our study were misdiagnosed by the peripheral health workers, severely affecting their quality of life due to delay in treatment and landing them up in post-herpetic neuralgia, a herpes zoster complication.

These cases highlight the significance of continuing to check vaccine safety while mass vaccination for the COVID-19 pandemic is ongoing, as well as the importance of documenting and communicating any vaccination-related adverse events, especially at the level of peripheral health workers. The need to enlist the help of atypical health professionals in acquiring data for the development of control strategies is emphasized.

## Materials and methods

We present a case series of 10 patients who presented with herpes zoster reactivation after COVID-19 vaccination, with two cases in detail focussing on the role of peripheral health worker. Demographic details, comorbidities, clinical features, history of COVID-19 vaccination, and treatment history were noted down, and pictures were taken after obtaining consent from the patients.

## Results

Case 1

**Figure 1:**
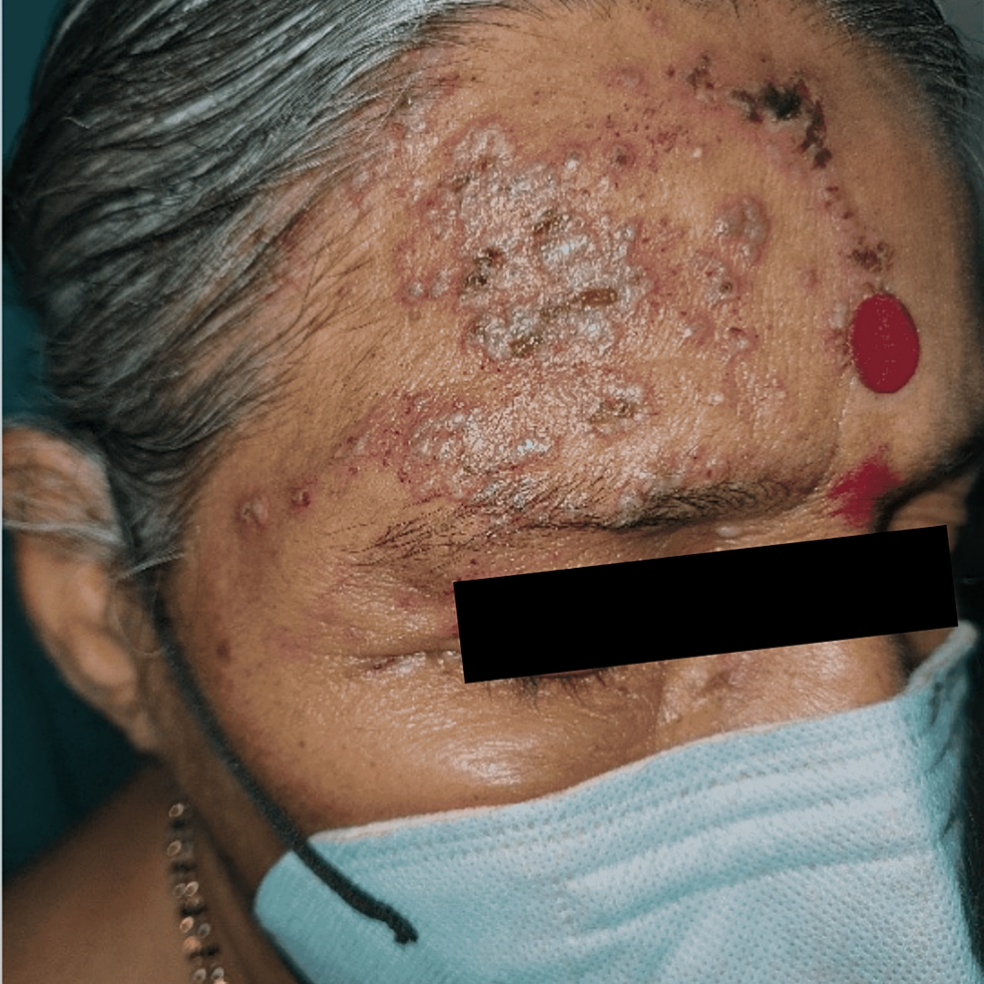
Multiple well-defined grouped vesicles overlying erythematous base with crusting over a few over the right side of forehead and scalp involving periocular area Dermatome C2

A 73-year-old woman went to her local community healthcare center complaining of painful skin rashes that had been bothering her for the past three days, starting with pain and burning sensations on the right side of her forehead and right eye, followed by multiple painful eruptions in the same area the next day. She was given a five-day course of 5 mg levocetirizine tablets at night and a topical steroid formulation for local application. Antivirals were not administered. According to the patient, no history of COVID-19 immunization was taken. After five days, she alerted the peripheral health worker that the pills were not providing any relief, and she was instructed to remain on the same medications for another five days. After ten days of treatment, she was still unsatisfied, which compelled her to visit our dermatology OPD because of the excruciating pain and burning sensation. Her medical history was taken, and she was examined thoroughly. She did not give any past history of similar complaints and has been a known case of diabetes mellitus and hypertension for 15 years, with regular medications. She had no previous history of COVID-19 infection, and she had had her first dose of COVID-19 immunization seven days before she presented to the peripheral health center. This history was overlooked by a peripheral health provider, who only provided symptomatic therapy without considering it to be a COVID-19 vaccination problem. Multiple clustered vesicles with crusting over an erythematous base were found on examination in the right frontotemporal area, spreading to the periocular area below; V1 dermatome was involved (Figure 1).

The patient was conscious and oriented, yet she was terrified and trembling. The rest of the physical examination went off without a hitch. She also denied suffering from headaches, blurred vision, chest discomfort, palpitations, gastrointestinal symptoms, urine changes, or limb weakness. Based on the characteristics of rash, the patient's typical signs and symptoms, and the context of being immunosuppressed due to her history of COVID-19 immunization, a diagnosis of herpes zoster was made. For five days, she was given 800 mg of acyclovir five times a day, 500 mg of paracetamol twice a day, and calamine lotion, which led to significant improvement. Ideally, antivirals should have been started as soon as she arrived at her local health center, as antivirals are most successful in treating herpes zoster if taken within 2-3 days of the commencement of symptoms. In her follow-up after two months, she still complained of pain at the issue spots. After being diagnosed with postherpetic neuralgia, she was prescribed a night-time dose of pregabalin.

Case 2

**Figure 2:**
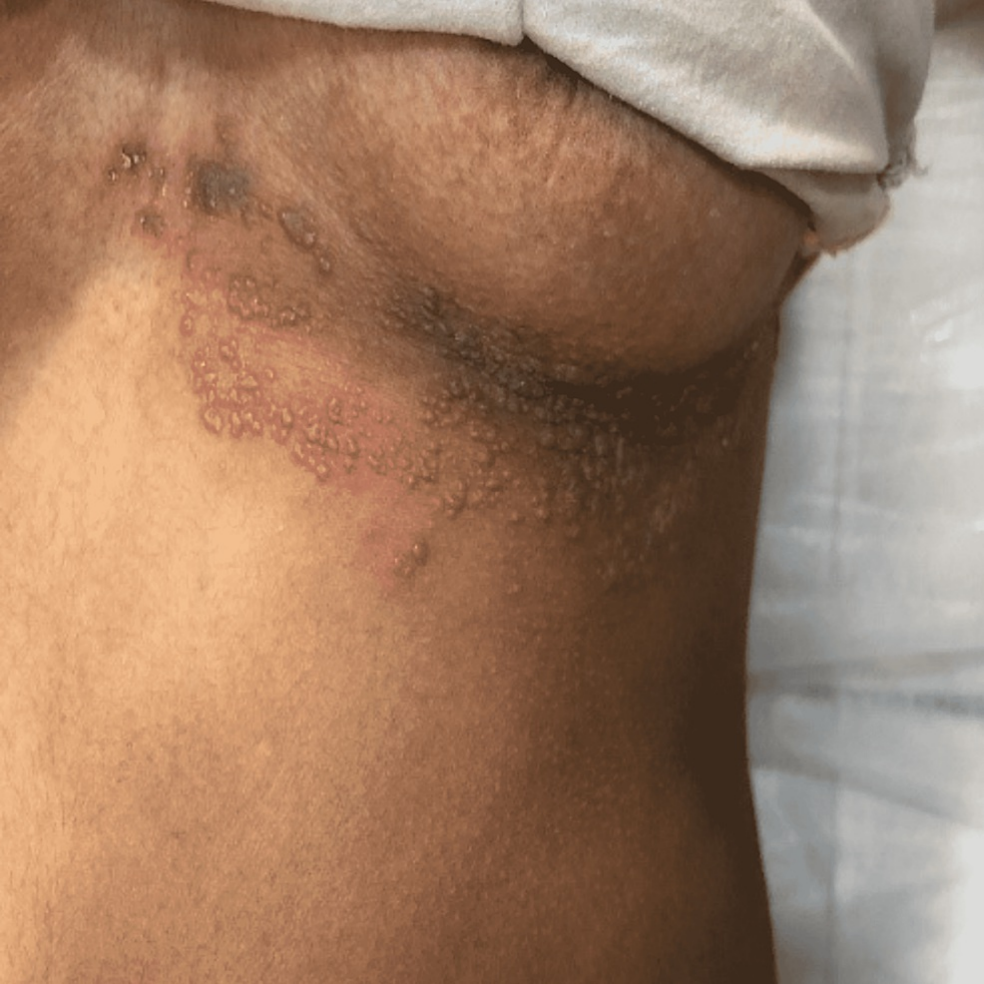
Multiple well-defined grouped vesicles overlying erythematous base present over the inframammary region of the left breast Dermatome T5-T6

Another 50-year-old woman, without any known comorbidity, complained of red burning fluid-filled eruptions grouped together below her left breast, extending up the axilla, for the past 15 days. The symptoms began four days after taking the first dose of the COVID-19 vaccine. She had no history of fever, limb weakness, or paraesthesia. On inspection, multiple well-defined erythematous scaly plaques were seen over the left inframammary region (Figure 2). It was the T5-T6 dermatome that was involved. She had a history of chickenpox infection at three years of age, which subsided without treatment. She did not have a history of any other comorbidity like diabetes, hypertension, and pulmonary tuberculosis. VZV reactivation was diagnosed, and antiviral drugs were prescribed to treat it. When asked about her medical history, she revealed that she had previously sought treatment at a number of peripheral clinics for a variety of ailments but with no relief. After a series of physically, mentally, and financially draining consultations, the patient, who was from a low socio-economic background, came to our OPD in a state of anguish.

Cases 3-10

Other patients' details are presented in Table 1.

**Table 1:** Details of the patients presenting with reactivation of varicella virus after COVID-19 vaccination VZV - varicella zoster virus

Age (years) /sex	Comorbidity	Dermatome involved	Dose of COVID-19 vaccination	Days of VZV activation after COVID-19 vaccination	Past status of varicella infection
32/M	None	Multiple vesicles grouped together over the erythematous base present over the left parasternal area and the left scapular area distributed along the T3-T4 dermatome.	1^st^ dose	10 days	20 years back
35/M	None	Multiple well-defined tense vesicles were present over the right frontotemporal area involving the ipsilateral scalp involving the C2 dermatome.	1^st^ dose	9 days	None
39/F	None	Multiple well-defined erythematous papules discretely placed of size around 0.5 cm diameter were present over the left side of the back over the infrascapular area extending anteriorly up to the mid-axillary line involving the T5-T6 dermatome.	2^nd^ dose	8 days	None
45/F	Diabetes mellitus	Multiple erythematous vesicles clustered together over the lateral aspect of the right thigh involving L4 dermatome.	1^st^ dose	2 weeks	Unknown
46/M	None	Multiple discrete vesicles with a few plaques over erythematous base situated along the scapular area and mid-axillary line - the right side involving T3-T4 dermatome.	1^st^ dose	10 days	None
75/F	None	Multiple well-defined erythematous scaly plaques were present over the right upper arm involving the C5 dermatome.	2^nd^ dose	3 weeks	None
50/M	None	Multiple well-defined vesicles with the erythematous base were present over the right side of the mid-back along the T6 dermatome.	1^st^ dose	5 days	None
60/F	None	Examination revealed multiple well-defined erythematous pruritic vesicles situated over the right hypochondrium along the T10-T11 dermatome.	1^st^ dose	7 days	None

In all of the cases, routine investigations (complete blood count, liver function test, kidney function test, urine routine and microscopic examination, serum electrolytes, thyroid profile, blood sugars, and glycosylated hemoglobin [HbA1C]) were conducted and found to be within normal limits. 

Only two of 10 patients gave a history of varicella infection in their childhood. Other patients were unable to recall properly any past history of varicella infection. None of our patients had any past episodes of herpes zoster. Two patients were known cases of diabetes mellitus and were on medication for the same. Their HbA1C was within normal limits. The rest of the patients were free of any associated comorbidity and were immunocompetent.

For a period of 5-7 days, all patients were given oral antiviral drugs: 1 gm of valacyclovir thrice daily or 800 mg tablet of acyclovir five times daily for five days, and symptomatic care with antihistamines, nonsteroidal anti-inflammatory drugs (NSAIDs), and topical antibiotics with calamine lotion. None of the patients had received a zoster virus vaccine. Patients were observed for two-three months. Two out of 10 patients developed postherpetic neuralgia after two months.

## Discussion

Herpes zoster (HZ) incidence rises with age, from 3.1 per 1000 person-years for those 45-54 years old to 5.7 for those 55-64 years old and 11.8 per 1000 person-years for those over 65 years old [8]. We present a group of ten HZ reactivation cases, six of which were females. Within three weeks of getting the first dose of the COVID-19 vaccine, they all suffered herpes zoster reactivation. The symptoms and clinical signs of reactivation after the COVID-19 vaccine were generally comparable to herpes reactivation. Increased age is the most important risk factor for VZV reactivation, but other risk factors include immunocompromised states, such as HIV infection, trauma, stress, or concomitant diseases including malignancy, hepatic or chronic renal disease [9].

Postherpetic neuralgia is the most common HZ consequence, followed by HZ ophthalmicus, acute retinal necrosis, Ramsay Hunt syndrome, Bell's palsy, aseptic meningitis, encephalitis, peripheral motor neuropathy, myelitis, Guillain-Barre syndrome, and bacterial skin infection [10]. In addition to pain, around 8-10% of HZ patients develop complications, and the recurrence rate is 10.96 per 1000 person-years [11]. Cell-mediated immunity is essential for maintaining latency and limiting the risk of reactivation, and recurrences are more likely in immunocompromised patients.

The COVID-19 infection produces an immunosuppressive state due to functional impairment and a concomitant quantitative decrease in T lymphocytes, particularly CD4+ T cells, CD8+ T cells, and natural killer cells which could be the plausible mechanism for the increased susceptibility to herpes zoster reactivation in COVID-19 patients [12]. Few studies have reported the reactivation of numerous other viruses, including human herpesvirus 6, 7, and Epstein-Barr virus, after COVID-19 infection [13].

To prevent this infection, several nations have begun vaccination with inactivated or live vaccines, and a variety of side effects have been observed following COVID-19 inoculation. Mild-to-moderate pain at the injection site, headache, and weariness characterize the safety profile of the COVID-19 mRNA vaccine, while the incidence of major, long-term side effects is still being assessed.

HZ after vaccination is a rare occurrence in the literature. Following inactivated influenza, hepatitis A, rabies, and Japanese encephalitis vaccines, Walter et al. documented three cases of herpes virus reactivation [14], while Bayas et al. described a case of herpes zoster following yellow fever vaccination [15]. Only minimal information about COVID-19 immunization is available. This case series is one of the few cases of herpes zoster after receiving inactivated COVID-19 vaccine that we are aware of. Given the recent availability of the COVID-19 vaccine, the fundamental reason for VZV reactivation in our circumstance is yet unknown. The occurrence of HZ within the time window 1-21 days after vaccination in our case series defined the increased risk and the observed involvement of T cell-mediated immunity suggesting that COVID-19 vaccination is a likely cause for the reactivation of latent VZV. None of our patients had any other risk factor for developing herpes zoster except the increasing age and diabetes mellitus in two patients, which was already under control. We could not be certain whether it was a de-novo infection or reactivation of herpes zoster as the majority of the patients were not able to recall their past medical history properly. 

At the time of writing, the United States Vaccine Adverse Event Report System (VAERS) has already reported 2512 cases of HZ (1.3%) of total reported events after tozinameran vaccine, 1763 cases (0.9%) after mRNA-1273, and 302 (0.7%) after AD26.COV2.S [16]. HZ is underreported as a post-immunization adverse event, thereby underestimating the incidence of post-COVID-19 vaccination sequelae.

Vaccination initiatives for COVID-19 are in full swing. Given the large number of people who will be vaccinated against SARS-CoV-2, a possible causal link could result in a large number of cases among the elderly, with devastating consequences. Post-marketing surveillance procedures must be in place, and ongoing vaccination safety assessments are critical for detecting any occurrence that could reduce the projected benefits and, as a result, taking obligatory action to reduce hazards amongst vaccinated people. HZ appears to be a "possible" yet uncommon adverse event following immunization (AEFI). Our findings necessitate a further investigation of the possible relationship between COVID-19 and herpes zoster in the context of vaccinating elderly and/or immunocompromised people.

In most impoverished nations, data for designing disease prevention initiatives is unavailable due to the high cost of gathering it. This study looked into using community key informants and peripheral health personnel to collect data on post-COVID-19 vaccination problems. Even in the poorest sections of the country, primary health care providers' prior knowledge can aid in the identification, quick treatment, and reporting of these potential problems following COVID-19 immunization. Antivirals work best if started within 2-3 days of the onset of HZ symptoms; therefore, the two individuals mentioned in our case series might not have ended up with postherpetic neuralgia if they had been diagnosed and managed promptly by peripheral health practitioners.

Fortunately, HZ is a manageable condition that is commonly associated with disability, particularly among the elderly, and existing therapy only affects the degree and length of pain, reduces viral shedding, and avoids postherpetic neuralgia. Because of the increased awareness of this complication, more HZ cases are being reported and communicated after the COVID-19 vaccination. Furthermore, these findings will help clinicians decide whether to give prophylactic valacyclovir before immunization for people who are at a higher risk of VZV reactivation after receiving the SARS-CoV-2 vaccine.

## Conclusions

The comprehensive evaluation will determine whether more should be done to raise awareness about potential dermatological complications such as VZV reactivation and how to remain prepared to manage them efficiently. However, the complication we report after the Covishield vaccination could just be a one-of-a-kind, isolated instance, and each person should have no hesitations about receiving the COVID-19 vaccine.
